# Continuous Ingestion of *Lacticaseibacillus rhamnosus* JB-1 during Chronic Stress Ensures Neurometabolic and Behavioural Stability in Rats

**DOI:** 10.3390/ijms23095173

**Published:** 2022-05-05

**Authors:** Agata Chudzik, Tymoteusz Słowik, Katarzyna Kochalska, Anna Pankowska, Artur Łazorczyk, Marta Andres-Mach, Radosław Rola, Greg J. Stanisz, Anna Orzyłowska

**Affiliations:** 1 Department of Neurosurgery and Paediatric Neurosurgery, Medical University of Lublin, Jaczewskiego 8, 20-090 Lublin, Poland; agata.chudzik@umlub.pl (A.C.); radoslaw.rola@umlub.pl (R.R.); stanisz@sri.utoronto.ca (G.J.S.); 2 Independent Laboratory of Cancer Diagnostics and Immunology, Department of Oncological Gynaecology and Gynaecology, Medical University of Lublin, Chodźki 4a, 20-093 Lublin, Poland; 3 Experimental Medicine Center, Medical University of Lublin, Jaczewskiego 8d, 20-090 Lublin, Poland; tymoteusz.slowik@o2.pl; 4 Department of Radiography, Medical University of Lublin, Staszica 16, 20-081 Lublin, Poland; kate123@onet.com.pl (K.K.); zubianna@gmail.com (A.P.); artur19o2@tlen.pl (A.Ł.); 5 Isobolographic Analysis Laboratory, Institute of Rural Health, Jaczewskiego 2, 20-090 Lublin, Poland; mandres@poczta.wp.pl; 6 Physical Sciences, Sunnybrook Research Institute, 2075 Bayview Avenue, Toronto, ON M4N 3M5, Canada; 7 Department of Medical Biophysics, University of Toronto, 101 College Street, Toronto, ON M5G 1L7, Canada

**Keywords:** *Lacticaseibacillus rhamnosus* JB-1, brain–gut–microbiome axis, depression, chronic unpredictable mild stress, magnetic resonance spectroscopy

## Abstract

The intestinal microbiome composition and dietary supplementation with psychobiotics can result in neurochemical alterations in the brain, which are possible due to the presence of the brain–gut–microbiome axis. In the present study, magnetic resonance spectroscopy (MRS) and behavioural testing were used to evaluate whether treatment with *Lacticaseibacillus rhamnosus* JB-1 (JB-1) bacteria alters brain metabolites’ levels and behaviour during continuous exposure to chronic stress. Twenty Wistar rats were subjected to eight weeks of a chronic unpredictable mild stress protocol. Simultaneously, half of them were fed with JB-1 bacteria, and the second half was given a daily placebo. Animals were examined at three-time points: before starting the stress protocol and after five and eight weeks of stress onset. In the elevated plus maze behavioural test the placebo group displayed increased anxiety expressed by almost complete avoidance of exploration, while the JB-1 dietary supplementation mitigated anxiety which resulted in a longer exploration time. Hippocampal MRS measurements demonstrated a significant decrease in glutamine + glutathione concentration in the placebo group compared to the JB-1 bacteria-supplemented group after five weeks of stress. With the progression of stress, the decrease of glutamate, glutathione, taurine, and macromolecular concentrations were observed in the placebo group as compared to baseline. The level of brain metabolites in the JB-1-supplemented rats were stable throughout the experiment, with only the taurine level decreasing between weeks five and eight of stress. These data indicated that the JB-1 bacteria diet might stabilize levels of stress-related neurometabolites in rat brain and could prevent the development of anxiety/depressive-like behaviour.

## 1. Introduction

There has been growing evidence that gut bacteria have an impact on brain neurometabolism via neuronal, endocrine, and immune mechanisms that altogether comprise the “brain–gut–microbiome” (BGM) axis [1,2]. Through the BGM axis, the brain affects the gut bacteria environment by modulating gut physiological status and composition. Conversely, intestinal microbiota modulates levels of neurotransmitters in the brain and, consequently, influences mood and behaviour [3,4,5,6]. Thus, the potential of probiotic bacteria as an alternate or adjuvant therapy in the prevention or treatment of anxiety and depression has been recognized [5,7]. The pathophysiology of several mental disorders, including anxiety and depression, is associated with alterations in brain metabolite levels [8,9]. Decreased levels in serotonin, dopamine, and noradrenaline levels and changes in glutamate and the γ-aminobutyric acid (GABA) cycle occurs in these, the principal excitatory and inhibitory neurotransmitters in the central nervous system (CNS) [3,8,9,10]. Moreover, perturbations in the hypothalamic-pituitary-adrenal (HPA) axis, neuroplasticity, neuroinflammatory and neurogenesis processes are involved in the pathophysiology of depression, and implicate the behavioural changes and disruption of cognitive abilities [11,12,13].

Several studies have shown that dietary supplementation with specific bacteria strains affects anxiety- and depression-like behaviours along with metabolic changes in the CNS [14,15,16,17]. Bacteria that have a beneficial effect on mental health when ingested in sufficient amounts are known as psychobiotics [18].

Previous studies have demonstrated the neuroactive properties of Lacticaseibacillus rhamnosus JB-1 (JB-1; recently reclassified from the *Lactobacillus* [19]) bacteria strain. In particular, oral, long-term supplementation with JB-1 changes the neurometabolites’ levels in mice and rats’ brains and mitigates the stress level as assessed with behavioural tests [14,15,20,21]. An in vivo study in BALB/c mice has shown an increase in the brain concentration of such amino acids as glutamate + glutamine (Glx), N-acetyl-aspartate (NAA), and GABA after supplementation with JB-1 bacteria [21]. Bravo et al. have demonstrated that dietary supplementation with JB-1 impacts GABAergic mechanisms, reduces stress induced corticosterone levels, and depression-like behaviour, while those changes are absent in vagotomized mice exposed to a probiotic diet [14]. This suggests that vagal signaling is critical for the effects of these bacteria, which has also been confirmed in a subsequent study examining the role of the vagal pathway in mediating neuronal response to JB-1 [22]. Additionally, it has been demonstrated that C57BL/6 mice fed with JB-1 attenuate behavioural deficits and immune changes in a chronic social defeat depression model [15]. Results obtained from our previous study [20] show that, in rats subjected to a chronic unpredictable mild stress (CUMS) and supplemented afterwards with JB-1 (interventional administration of bacteria), the levels of hippocampal GABA, glutamate, glutamine + glutathione, Glx, total creatine, and total N-acetylaspartate are restored (as measured by an in vivo magnetic resonance spectroscopy, MRS) and are comparable to the non-stressed, controlled animals. In addition, feeding the rats with JB-1 results in a reduction of stress-induced behaviour [20].

In the present work, we investigated whether treatment with *Lacticaseibacillus rhamnosus* JB-1 bacteria with simultaneous exposure to chronic stress could alter brain metabolites’ levels and behaviour. To achieve this goal, a rat model of the depressive-like disorder, behavioural study and in vivo MRS and Magnetic Resonance Imaging (MRI) were used. Rats were subjected to a CUMS protocol and were fed simultaneously with probiotic *L. rhamnosus* JB-1 diet or placebo. Before and after five and eight weeks of the procedure, animals were subjected to behavioural tests with the use of elevated plus maze (EPM). At the same time, MRS and MRI of the hippocampal area were performed to assess cerebral metabolite levels and tissue hydration, respectively. We hypothesized that the JB-1 bacteria provide a protective impact on neurochemical changes induced by chronic stress.

## 2. Results

### 2.1. Body Weight Gain

**Figure 1:**
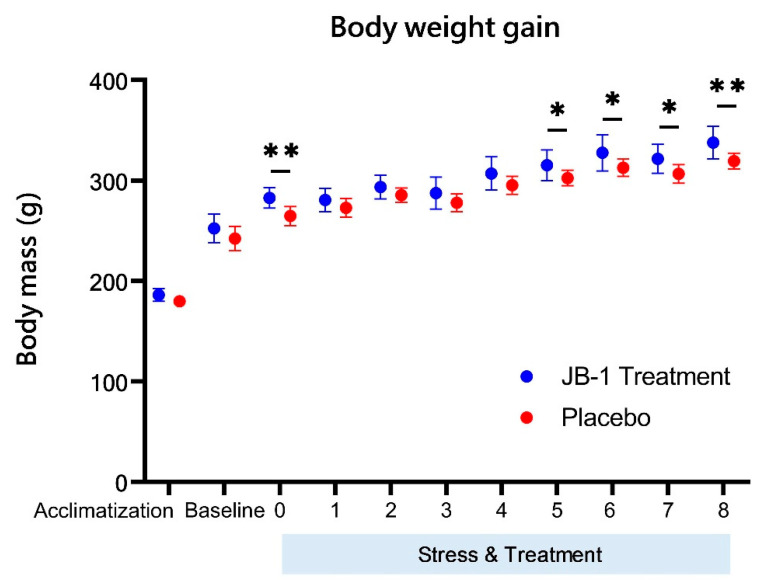
Rats body weight measured at the start of acclimatization, at baseline (before first MRI + MRS scanning), at week 0 (the start point of stress and feeding, after one week of MRI + MRS scanning) and every week during the stress protocol and the administration of JB-1 (N = 10) or placebo (N = 9). Data are represented as means ± SD. * *p* < 0.05; ** *p* < 0.01, two-way ANOVA analysis for repeated measures with post-hoc Duncan’s test.

Figure 1 presents the weight gains of JB-1-treated and placebo groups during the whole experiment. The animals’ body mass was similar at the beginning of the study, independent of the group (186 ± 6 g vs. 180 ± 4 g, means ± SD). The two-way ANOVA analysis for repeated measures confirmed the significant body weight gain throughout time for all the animals (time effect, F = 177.84, *p* < 0.0001); however, for the placebo group this gain was significantly slower (group effect, F = 8.21, *p* = 0.01). The differences occurred after one week of MRI scanning (between baseline and week 0) (283 ± 10 g for JB-1 group vs. 264 ± 10 g for placebo group, *p* = 0.006). The further divergences in the weight gain were observed after five weeks of CUMS and treatment, and continued until the end of the experiment (the last four measurements, after week 5: 315 ± 15 g vs. 302 ± 8 g, *p* = 0.04; after week 6: 328 ± 17 g vs. 312 ± 8 g, *p* = 0.02; after week 7: 322 ± 14 g vs. 304 ± 9 g, *p* = 0.01; and after week 8: 338 ± 16 g vs. 318 ± 9 g, *p* = 0.003).

### 2.2. Evaluation of Anti-Anxiolytic Behaviour

**Figure 2:**
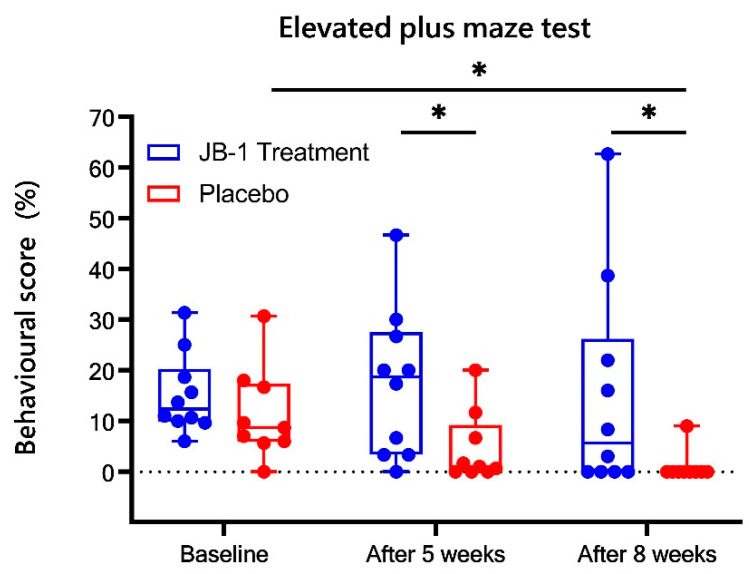
A ratio of time spent in the open arms of the maze (%) as a quantitative measure of behavioural score in the Elevated Plus Maze (EPM) behavioural test results in JB-1 treatment (N = 10) and placebo (N = 9) groups at baseline and after five and eight weeks of the stress protocol. Data are presented as median ± min/max. * *p* < 0.05, two-way ANOVA analysis for repeated measures with post-hoc Duncan’s test.

Each animal’s behaviour was assessed with an Elevated Plus Maze (EPM) test as a ratio of time spent in the open arms of the maze (%) and the results are presented in Figure 2. The baseline results were similar for both, JB-1 and placebo groups and showed no statistical difference (15.2 ± 2.3% vs. 11.4 ± 2.9%). The non-parametric ANOVA analysis showed a significant time effect (χ^2^ = 12.8, *p* = 0.002): rats treated with placebo demonstrated decrease of behavioural score after five weeks as compared to baseline (but not significant, *p* = 0.1), while after eight weeks of stress, placebo group animals exhibited almost total avoidance of exploration of the maze (*p* = 0.02 vs. baseline). In the JB-1 group, administration of JB-1 bacteria maintained the behavioural score at similar levels throughout the whole experiment. This led to significant differences between groups after five weeks of the CUMS protocol (17.4 ± 4.4% in JB-1 treated group vs. 4.6 ± 2.2%, *p* = 0.03), and after eight weeks of CUMS and diet supplementation (15.1 ± 6.3% vs. 1.0 ± 0.9%, *p* = 0.03) (significant group effect in two-way ANOVA: F = 5.17, *p* = 0.04).

### 2.3. Hippocampal Water Content Change Based on MRI Measurements

There has been strong scientific evidence that prolonged stress contributing to depression induces hippocampal shrinkage [11,23,24,25]. Such reduction of hippocampal volume is associated with diminished neuroplasticity and neurogenesis in this structure [11], as well as reduced water content as revealed by MRI in terms of longitudinal relaxation time T_1_ [23]. The reciprocal water content 1/W is proportional to longitudinal relaxation rate, R_1_ = 1/T_1_ (where T_1_ is the longitudinal relaxation time) in cerebral tissue [26]. In this study we measured R_1_ in the hippocampus to assess the influence of stress on the tissue water content.

**Figure 3:**
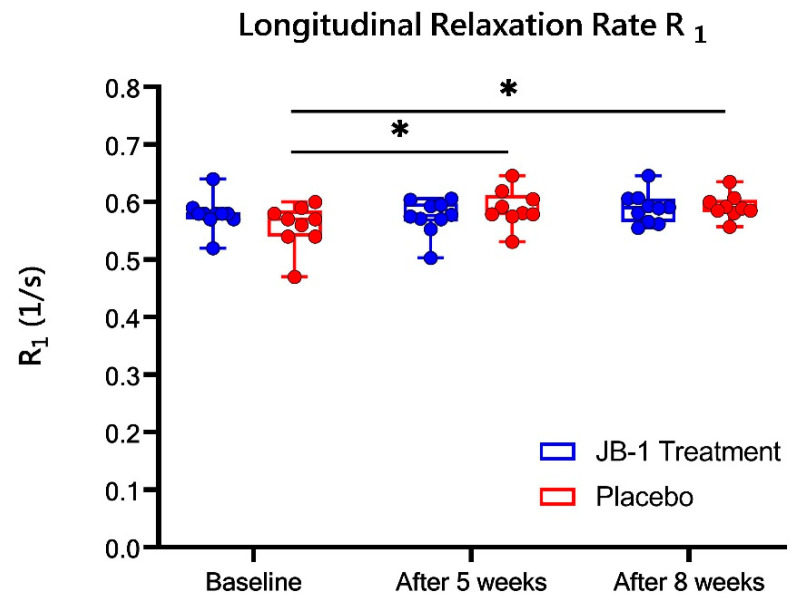
Longitudinal relaxation rate R_1_ in JB-1 (N = 10) treatment and placebo (N = 9) groups at baseline and after five and eight weeks stress protocol. Data are presented as median ± min/max. * p < 0.05, one-way Friedman’s ANOVA (χ^2^ statistic) analysis for determination of time effect.

The non-parametric ANOVA analysis showed significant changes of R_1_ over time (χ^2^ = 6.2, *p* = 0.04). Particularly, in the placebo group the increased R_1_ was observed after five weeks of CUMS (0.59 ± 0.01 s^–1^ vs. 0.56 ± 0.01 s^–1^ in baseline, *p* = 0.04) and also after eight weeks of stress (0.59 ± 0.01 s^–1^ vs. baseline, *p* = 0.03), Figure 3. In the JB-1 group no differences in R_1_ between three timepoints were observed.

### 2.4. MRS Data Quality and Fitting Accuracy

The hippocampal levels of brain neurometabolites were assessed with Magnetic Resonance Spectroscopy. From all the measured MRS data one subject from the placebo group exhibited insufficient signal quality, which led to the over- or underestimation of certain metabolites. Thus, this one case was excluded from the experimental population, and the resulting numbers taken into account were: JB-1, N = 10 and Placebo, N = 9. For the remaining data, the full width at half-maximum (FWHM) of the water line was, on average, 8.8 ± 0.6 Hz. The analyzed set of data exhibited very good quality with mean signal-to-noise ratio, SNR = 37 ± 4. Among all fitted metabolites the following 11 resulted in Cramer-Rao lower bounds (CRLB) < 25% (Table 1): choline (Cho), creatine (Cr), γ-aminobutyric acid (GABA), glutamate (Glu), glutamine (Gln), glutathione (GSH), myo-Inositol (m-Ins), N-acetylaspartate (NAA), N-acetylaspartylglutamate (NAAG), phosphocreatine (PCr), taurine (Tau), as well as macromolecular content (MM), and these metabolites were used for all further statistical analyses. The following numbers of outliers were also detected within certain metabolites: GABA^5weeks^ (*n* = 1), Gln^5weeks^ (*n* = 1), GSH^5weeks^ (*n* = 1), m-Ins^5weeks^ (*n* = 1), tCho^5weeks^ (*n* = 1), Gln^8weeks^ (*n* = 1) in placebo group, and m-Ins^baseline^ (*n* = 1) in JB-1 group, and these data were also excluded from comparisons.

**Figure 4:**
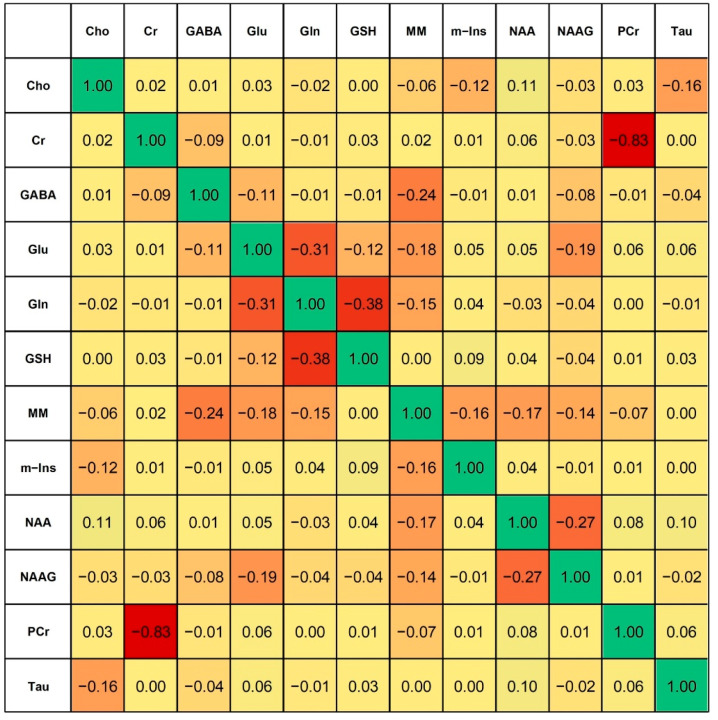
Correlations between neurometabolites quantified in vivo by MRS.

The heat map of correlations between metabolites is presented in Figure 4. Significant negative correlation was observed between: Glu and Gln (r = −0.31), Gln and GSH (r = −0.38), Cr and PCr (r = −0.83), NAA and NAAG (r = −0.27). Due to statistically significant correlations, these metabolites were combined into sums: Glx (Glu + Gln); Gln + GSH; total creatine: tCr (Cr + PCr); total NAA: tNAA (NAA + NAAG), and also total choline: tCho as sum of Cho and phosphorylcholine (PCho), as usually reported in other works. Since the correlations between Glu and Gln, as well as between NAA and NAAG were weak, these metabolites were additionally presented separately.

### 2.5. MRS-Based Concentrations of Neurometabolites—Standard Approach of Data Analysis

**Figure 5:**
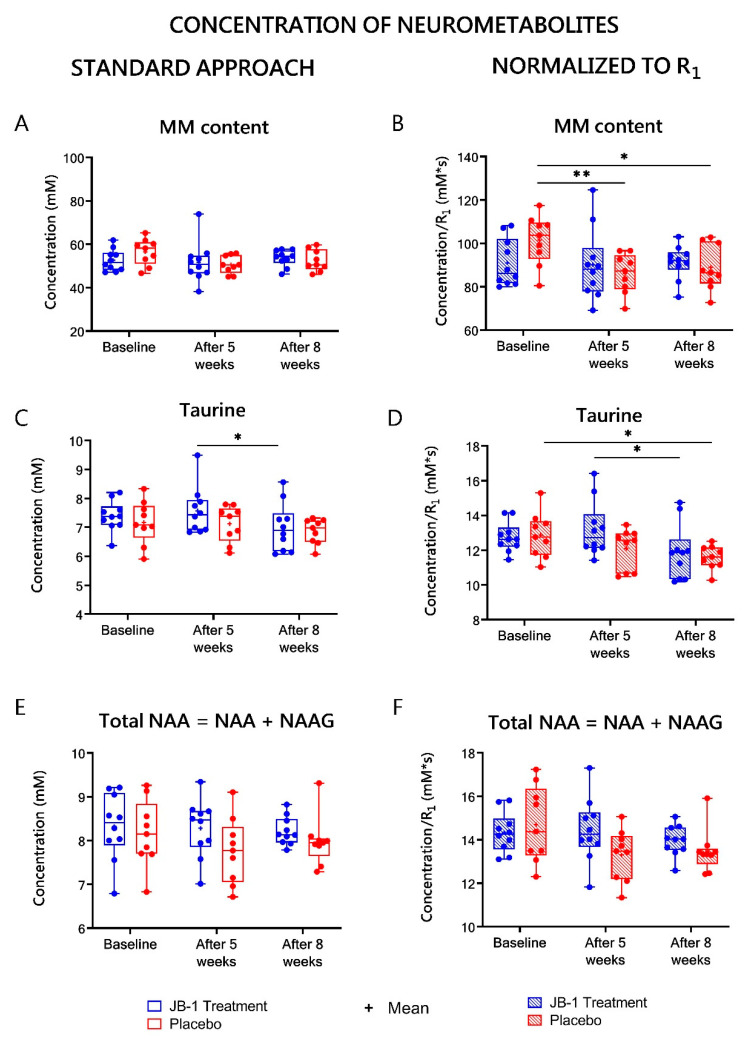
Hippocampal MM content (**A**), taurine (**C**), tNAA (**E**) levels calculated using typical water referencing (standard approach)—left column. Hippocampal MM content (**B**), taurine (**D**), tNAA (**F**) levels normalized to R_1_—right column. Neurometabolites’ concentrations were assessed by MRS in JB-1 treatment and placebo groups at baseline and after five and eight weeks stress protocol. Data are presented as median ± min/max. * *p* < 0.05; ** *p* < 0.01, two-way ANOVA analysis for repeated measures with post-hoc Duncan’s test.

**Figure 6:**
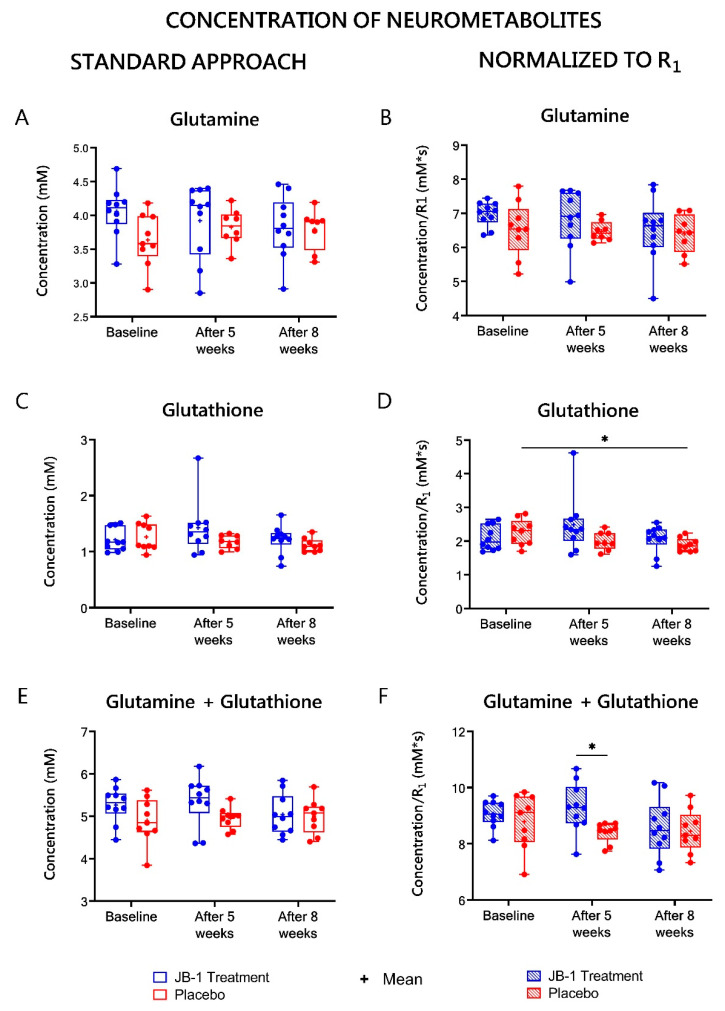
Hippocampal glutamine (**A**), glutathione (**C**), and glutamine + glutathione (**E**) levels calculated using typical water referencing (standard approach)—left column. Hippocampal glutamine (**B**), glutathione (**D**), and glutamine + glutathione (**F**) levels normalized to R_1_—right column. Neurometabolites’ concentrations were assessed by MRS in JB-1 treatment and placebo groups at baseline and after five and eight weeks stress protocol. Data are presented as median ± min/max. * *p* < 0.05; two-way ANOVA analysis for repeated measures with a post-hoc Duncan’s test.

**Figure 7:**
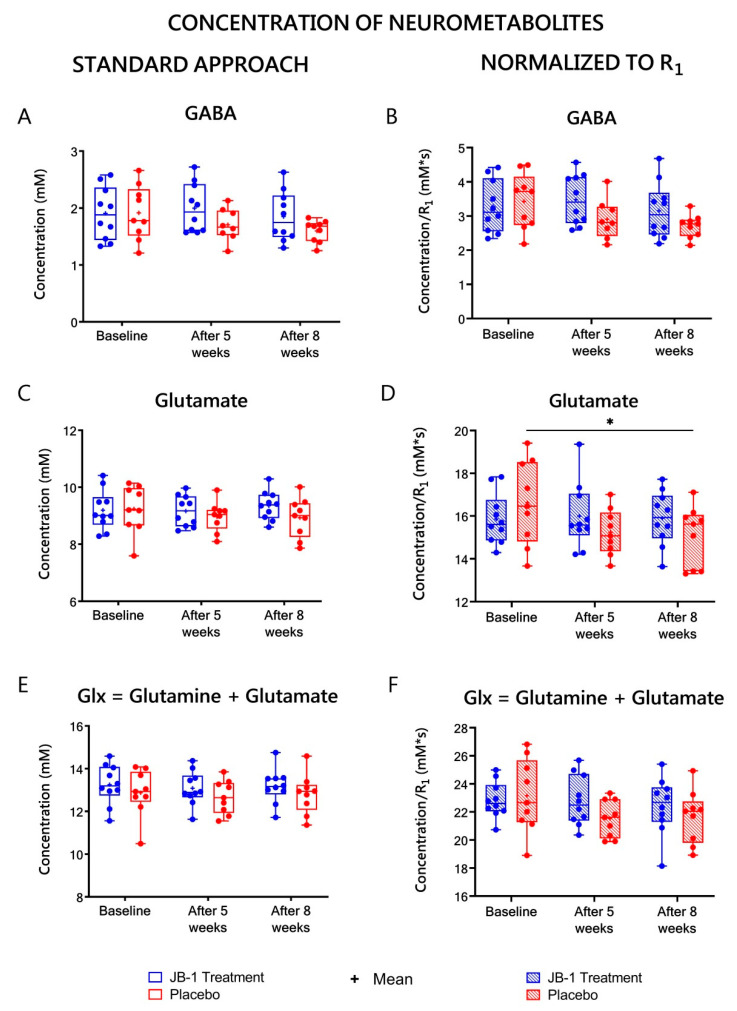
Hippocampal GABA (**A**), glutamate (**C**), and Glx (**E**) levels calculated using typical water referencing (standard approach) are shown in the left column. Hippocampal GABA (**B**), glutamate (**D**), and Glx (**F**) levels normalized to R_1_—are shown in the right column. Neurometabolites’ concentrations were assessed by MRS in the JB-1 treatment and placebo groups at baseline and after five and eight weeks stress protocol. Data are presented as median ± min/max. * *p* < 0.05; two-way ANOVA analysis for repeated measures with post-hoc Duncan’s test.

The concentrations of all the metabolites calculated with standard water referencing (assuming, that gray matter water content is 80% [27]) are listed in Supplementary Table S1 and Figure 5, Figure 6 and Figure 7 (left columns). From all the neurometabolites assessed with this approach, only Tau showed statistically significant changes over time as analyzed by two-way ANOVA (F = 4.24, *p* = 0.02). In post-hoc analyses a statistically significant difference in Tau levels in the JB-1 group between five and eight weeks of treatment (*p* = 0.02) was found (Figure 5C). There has also been observed a decreasing trend in time in m-Ins, but it failed to reach statistical significance (F = 2.84, *p* = 0.07). The only neurometabolites showing a tendency in between subject effect were Gln + GSH (F = 3.86, *p* = 0.07) Figure 6E.

### 2.6. MRS-Based Concentrations of Neurometabolites Normalized to R_1_

To eliminate the influence of subject-dependent hydration status of hippocampal tissue and changes in hydration between baseline, five-week and eight-week stress, the MRS-derived results were corrected to R_1_ values estimated form MRI. The results of metabolite concentrations corrected for a water content (normalized to R_1_) are presented in Supplementary Table S2 and Figure 5, Figure 6 and Figure 7 (right columns). The baseline values for the JB-1 and placebo treated groups for neurometabolites showed no statistically significant differences. A significant group effect was observed in two-way ANOVA assessment of the Gln + GSH level (F = 7.7, *p* = 0.005). The post-hoc analysis showed significantly lower levels of Gln + GSH in the placebo group relative to JB-1 group measurements after five weeks of stress (8.4 ± 0.1 vs. 9.3 ± 0.3 mM*s, *p* = 0.01) (Figure 6F), but this difference diminished at week eight. After five weeks of stress in the placebo group, the slight decrease of the following metabolites were observed vs. JB-1 levels: GABA, GSH, Glx, NAA, tNAA, and Tau, but did not meet statistical significance (Table S2, Figure 5, Figure 6 and Figure 7).

The ANOVA analysis for repeated measures indicated a significant time × group effect for GSH (F = 3.8, *p* = 0.04) and a diminishing trend in the time effect for this metabolite was observed (F = 3.5, *p* = 0.05). After eight weeks of CUMS protocol, in the placebo group, GSH decreased by 8% as compared to baseline (from 2.3 ± 0.1 to 1.9 ± 0.1 mM*s, *p* = 0.045) (Figure 6D). A significant time effect was observed for Tau (F = 5.49, *p* = 0.008). After eight weeks of CUMS protocol Tau decreased by 10% as compared to baseline in placebo group (from 12.9 ± 0.4 to 11.6 ± 0.2 mM*s, *p* = 0.02) (Figure 5D). In turn, in the JB-1 group, there was a significant difference in Tau level between weeks five and eight of treatment (decrease from 13.3 ± 0.5 to 11.9 ± 0.5 mM*s, *p* = 0.02, Figure 5D). MM level also showed a statistically significant time effect (F = 7.05, *p* = 0.03). After five weeks of stress in the placebo group, the MM level was significantly lower compared to a baseline measurement (86.1 ± 2.9 vs. 101.5 ± 3.6 mM*s, *p* = 0.008), and this low level was maintained for three more weeks (88.8 ± 3.3 mM*s, *p* = 0.02 vs. baseline) (Figure 5B). Glu, NAA, and tNAA in the placebo group also showed a decreasing trend after five weeks of stress compared to baseline, but did not reach statistical significance. The post-hoc analysis after eight weeks of stress compared to baseline showed a significant decrease of 9% in Glu level (15.1 ± 0.4 vs. 16.6 ± 0.6 mM*s, *p* = 0.04) (Figure 7D) and a diminishing trend by 21% in GABA (Figure 7B), 9% in NAA, and 8% in tNAA (Figure 5F) in the placebo group, but these changes failed to reach statistical significance (Table S2). Besides taurine, none of the assessed metabolites in the JB-1 group showed changes throughout the whole experiment.

In the next step, the JB-1 treated group was divided into two additional groups according to behavioral results observed after five and eight weeks of stress: the group of “Positive response” (behavioral score > 0), and the one of “Negative response” (behavioral score = 0). The MRS data from such groups are presented in Table 2 as means ± SD. After five weeks of stress only one subject from JB-1 treated animals exhibited a negative response. Based on a s student’s t-test, the “Positive response” JB-1 group (N = 9) displayed significantly higher concentrations of Gln + GSH (9.3 ± 0.9 vs. 8.4 ± 0.4 mM*s, *p* = 0.01), NAA (14.4 ± 1.3 vs. 12.9 ± 1.2 mM*s, *p* = 0.02) and tNAA (9.3 ± 0.9 vs. 8.4 ± 0.4 mM*s, *p* = 0.02) as compared to the placebo group (N = 9).

After eight weeks of stress in the “Positive response” JB-1 group (N = 6), the levels of tCho, GABA, Glx and tNAA were significantly higher than in the placebo group (N = 9) (2.7 ± 0.3 vs. 2.3 ± 0.3 mM*s, *p* = 0.02; 3.5 ± 0.8 vs. 2.7 ± 0.3 mM*s, *p* = 0.02; 23.4 ± 1.5 vs. 21.2 ± 1.5 mM*s, *p* = 0.04; and 14.2 ± 0.6 vs. 13.2 ± 0.5 mM*s, *p* = 0.01; respectively) as assessed by one-way ANOVA analysis with post-hoc Duncan’s test. Moreover, the “Positive response” JB-1 group exhibited significantly higher values than the “Negative response” JB-1 group (N = 4) in the following neurometabolites concentrations: tCho (2.7 ± 0.3 vs. 2.2 ± 0.1 mM*s, *p* = 0.005), GABA (3.5 ± 0.8 vs. 2.6 ± 0.5 mM*s, *p* = 0.02), Gln + GSH (9.1 ± 0.9 vs. 7.8 ± 0.8 mM*s, *p* = 0.02), and Glx (23.4 ± 1.5 vs. 20.9 ± 1.9 mM*s, *p* = 0.03).

## 3. Discussion

In the present study, we demonstrated the influence of the administration of *Lacticaseibacillus rhamnosus* JB-1 bacteria given simultaneously with chronic stress on behaviour, weight gain, and hippocampal neurometabolites’ levels changes. The behavioural test combined with in vivo MRS showed that the JB-1 bacteria stabilize the levels of stress-related neurometabolites in the rat brain and prevent the development of anxiety/depressive-like behaviour. It confirmed and expanded upon previous findings demonstrating behavioural and metabolic alterations in rodents subjected to chronic stress and treated with probiotic bacteria.

Baseline body mass was similar in both groups, and as the stress protocol progressed, the placebo group gained weight, as expected [20,28,29], at a slower rate than the bacteria-supplemented group. Moreover, the depressive-like disorder was already developed at week 5 of the CUMS protocol, which was particularly reflected in body mass differences between groups at week 5 (Figure 1). This behavioural endpoint in the CUMS model has also been reported by Hu et al. [30]. The supplementation with JB-1 bacteria ensured more stable weight gain in animals despite the stressing factors, and along with behavioural measures suggested that the probiotic diet increased the animals’ resistance to stress.

In the behavioural elevated plus maze (EPM) test, there were no noticeable differences between groups in baseline behaviour. After five and eight weeks of stress, the rats treated with *Lacticaseibacillus rhamnosus* JB-1 exhibited a greater interest in exploring open arms of the EPM compared to the placebo group, which indicated a preventive effect of JB-1 on the development of anxiety symptoms. These data supported other literature findings [14,15,17,31] and those of our previous study [20], suggesting that supplementation with specific bacterial strains reduces anxiety- and depressive-like behaviours.

In this study two different approaches of hippocampal metabolite quantification based on an MRS measurement were used. The standard approach, typical for many MRS-based studies, assumed that tissue water content was constant, and in gray matter it was evaluated at approximately 80% [27]. Such assumption typically enables the quantification of metabolite concentrations in mM, and allows for the comparison of results between different studies. This approach was also used in our previous study on CUMS models treated with JB-1 under a different paradigm: after the depressive-like disorder was already developed [20]. In the current study, the baseline concentrations of hippocampal metabolites assessed with the standard approach were, in general, slightly higher than in our previous research [20] (Glu: 9.2 ± 0.2 mM now vs. 8.7 ± 0.7 mM previously; Gln + GSH: 5.1 ± 0.2 mM now vs. 4.8 ± 1.1 mM previously; tNAA: 8.2 ± 0.2 mM now vs. 7.3 ± 0.5 mM previously), which could be attributed to age differences (six weeks vs. 14 weeks) [32,33]. In the previous study much older rats were used (eight week difference for baseline scans) [20]. Age differences between our two studies could also contribute to less pronounced stress effect on the metabolite level changes in the placebo group, as it was shown that earlier onset of depressive disorder results in less severe brain alterations [34], and brain metabolites naturally change with age, as well [32,33]. With the standard method of data analysis, we found in the placebo group a statistically significant decrease in MM concentration after five weeks of stress compared to baseline results and a decrease in taurine levels in the JB-1 supplemented group between five and eight weeks of stress. Finally, a difference of the study could also stem from different paradigms (continuous, eight-week aggressive stress in the present study vs. post five-week stress in the previous study).

It should be noted, however, that hippocampal hydration was changing throughout the experiment, as evidenced in R_1_ measurements (Figure 3). In the placebo group, R_1_ increased after five weeks of stress (0.59 ± 0.01 s^–1^ vs. 0.56 ± 0.01 s^–1^ in baseline), and after three more weeks this value was still higher compared to baseline (0.59 ± 0.01 s^–1^ vs. baseline). The significant progressive increase of R_1_ values in the placebo group indicated that the percentage water content in the hippocampus decreases due to chronic stress, which was in accordance to other studies of depressive disorders [23]. Such a change is generally associated with hippocampal shrinkage due to over-stimulation by prolonged continuous stress, the phenomenon of which is observed in depressive cases [24,25]. This was not observed in the JB-1 treated group, in which R_1_ values were stable during the eight-week timespan. In our previous study we did not observe the R_1_ differences between pre- and post-treatment timepoints in placebo treated CUMS rats [20]. That might be because in older animal’s water content changes due to ageing are slower [27]. Such confirmed stability of hippocampal water levels has justified the calculation of absolute amounts of metabolites in the standard procedure using a water reference signal, and assuming its constant concentration in our previous work [20]. In the present study, the whole experimental timespan covered the transition from adolescent to the adult stage of animal life. It has been shown that in such stage the cerebral water content drops by about 8–10% [35]. Interestingly, in the studies, where longitudinal brain metabolomics is investigated with the use of MRS, the water content change correction is frequent practice [35,36,37], but some authors explicitly assume that after six weeks of the birth the water content is not changing in healthy rat brain [37], or do not arise this aspect [38]. Based on our findings on R_1_ changes, a second, corrected analysis of MRS data was performed by normalizing the metabolites’ concentrations to the measured R_1_ to diminish the impact of differences in hippocampal water level.

In R_1_-normalized results from the stressed, placebo administrated group, a decrease in macromolecular content after five weeks of stress compared to baseline results was observed. These changes persisted after an additional three weeks of stress. Moreover, after eight weeks of CUMS, a decrease in taurine and glutathione concentrations were observed. The observed alterations in the stressed group, besides MM content level, were consistent with our previous study [20]. A previous report also shows that the five week CUMS protocol results in a reduction in total creatine, glutamate, glutamine + GSH, Glx, and tNAA levels versus baseline, which have not been observed to be significant in the placebo group of the present study [20].

Decreased levels of macromolecular content, MM has also been observed in other chronic stress rat model studies [39]. Macromolecular content corresponds to proteins and lipids profiles [40]. The diminished levels in the MM concentration in the placebo group after the CUMS procedure could be related to cellular stress or alterations in the brain lipidome [39,41,42].

While the stress protocol caused a decrease in the rat brain metabolites levels, simultaneous administration of JB-1 bacteria had a stabilizing effect on their concentration. As well, other studies investigating *Lacticaseibacillus rhamnosus* JB-1 indicate its involvement in central nervous system response [14,22]. In the present research, bacteria supplementation maintained Glu + GSH levels comparable to baseline measurements. Moreover, after five weeks of stress, the level of glutamine + glutathione was significantly higher in the JB-1 group than this observed in the placebo group. Considering the reduced GSH levels in the stressed, placebo-treated group, the stabilization of GSH in the JB-1 group may indicate a protective role of the bacteria to prevent oxidative stress, so that GSH was not used up [43]. Protective effects against oxidative stress are observed also in other studies investigating lactic acid bacteria [43,44]. Moreover, a significant decrease in Glx, N-acetylaspartate, and total N-acetylaspartate levels were observed in the non-treated group vs. the bacteria treated group positively responding in behavioral tests after five weeks of stress (Table 2), and for GABA, tCho, Glx and tNAA after eight weeks of stress as compared to the “Positive response” JB-1 group (Table 2). Stress-induced reductions in the concentrations of N-acetylaspartate and total N-acetylaspartate have been reported in other MRS-based studies of the hippocampus [45,46]. Such a decrease usually reflects a compromise of neuronal integrity, as these metabolites are interpreted as neuronal density and function markers [47]. Increased total choline level in the “Positive response” JB-1 group may be related to the stabilization of cellular lipid membranes [48]. The disruptions of glutamatergic metabolism in the stressed animals is also described in the literature [20,46,49], and it was observed in present study in placebo group as compared to the positively responding JB-1 group. Moreover, some reports describe stress-induced increases in the concentration of GABA [39,50] and Glu [51,52], on the other hand, others report that stress decreases these neurometabolites level [46,49]. The increased levels of such metabolites in the positively responding JB-1 group indicate that the supplementation with probiotic bacteria prevented these pathological, stress-induced changes in the hippocampus. A significant difference in taurine level was observed between the fifth and eighth week of bacteria supplementation. At week eight, taurine levels decreased compared to the results observed after five weeks of simultaneous bacteria supplementation and stress but were still higher than those observed in the placebo group. Bacterial supplementation during five weeks of stress influenced taurine level stabilization compared to the baseline result, but progressive stress and aging-related changes could have affected its concentration later in the experiment, as observed in other studies related to brain development [53,54,55,56]. The other metabolites showed no statistically significant differences between the initial results and the results after simultaneous stress and administration of bacteria. Administration of the bacteria prevented the reduction of metabolites concentrations in the rats’ brains which occurred in the placebo group. In our previous study, we have demonstrated that in the CUMS rat model, JB-1 bacterial supplementation after stress cessation brings back to normal the levels of glutamate and glutathione, glutamine + glutamate, GABA, total N-acetylaspartate, and glutamine + GSH in rat hippocampus [20]. Comparing these findings to our present study, it seems that no matter the paradigm that is investigated (post-stress bacterial treatment, or feeding with JB-1 simultaneously with stress), the JB-1 bacteria influence the similar cerebral metabolites with a beneficial, stabilizing effect. This suggests that the anti-anxiety and anti-depressant effect was comparable in both studies.

In summary, the *Lacticaseibacillus rhamnosus* JB-1 bacteria strain administration continued simultaneously with the chronic stress influenced stabilization of glutamate, glutathione, taurine and macromolecular content in the rat hippocampus and prevented the development of anxiety/depressive-like behavior.

## 4. Materials and Methods

### 4.1. Animals

All animal procedures were approved by the Local Ethical Committee for Animal Experiments, University of Life Sciences in Lublin (protocol code 62/2016, approved on 24 October 2016) and performed in accordance with the “Guide for the Care and Use of Laboratory Animals” (National Research Council (US) Committee for the Update of the Guide for the Care and Use of Laboratory Animals, National Academies Press (US), 2011).

Twenty male Wistar rats (180–200 g) were obtained from the Centre of Experimental Medicine, Medical University of Lublin, Poland. The animals were maintained under standard laboratory conditions (50 ± 5% humidity, 12 h light/dark cycle, 22 ± 1 °C) with free access to water and a laboratory chow diet (type Altromin 1324 IRR, Altromin International, Lage, Germany). Rats were housed in polypropylene cages for two weeks of acclimatization prior to the experiment. They were single-housed to avoid aggressive behaviours as a consequence of prolonged stress.

### 4.2. Experimental Procedure

After acclimatization, the animals were randomized into two groups: fed with probiotic JB-1 diet (JB-1, N = 10) or placebo (Phosphate Buffered Saline, PBS, N = 10). During the study, one subject from the placebo group was rejected due to insufficient MRS data quality, which led to group size reduced to N = 9. The rats were supplemented with JB-1 bacteria or PBS daily for eight weeks and simultaneously subjected to a chronic unpredictable mild stress (CUMS) protocol. Each rat underwent an elevated plus maze test of anxiety and cerebral magnetic resonance spectroscopy of the hippocampus region three times: before starting the CUMS protocol and dietary supplementation (baseline measurements), after five and after eight weeks of stress and dietary supplementation. The body weight of animals was measured every week. Following the last MRS, rats were euthanized by decapitation while under anesthesia.

### 4.3. Chronic Unpredictable Mild Stress Protocol

A chronic unpredictable mild stress (CUMS) procedure was performed similarly to that previously described by Hu et al. [30]. Rats were chronically exposed to seven different stressors: overnight illumination, five minutes cold swimming (at 4 °C), 24-h food deprivation, 24-h water deprivation, four hours of 45° cage tilt, 50 min in a cold environment (at 4 °C) and 24 h in a wet cage. Rats received one of these stressors randomly per day for eight weeks.

### 4.4. Probiotic Preparation and Treatment

*Lacticaseibacillus rhamnosus* JB-1 material was obtained from Alimentary Health Ltd., Co. (Cork, Ireland) and prepared as described previously [20]. Bacteria were suspended in De Man, Rogosa and Sharpe medium (MRS broth; Difco Laboratories, Detroit, MI, USA), divided in 1 mL aliquots, and frozen and stored at −80 °C before use. Each aliquot was thawed at room temperature and spun down in a microcentrifuge for 10 min at 2400 rpm. The bacteria pellets were then washed with sterile PBS three times and re-suspended in 1.2 mL sterile PBS. Each animal from the JB-1 group received 0.2 mL of bacteria suspension in PBS (1.7 × 10^9^ CFU) daily by oral gavage. The rats from the placebo group were gavaged with 0.2 mL of PBS daily.

### 4.5. Behavioural Study

The elevated plus maze test (EPM) was used to perform behavioral assessment. The EPM was described in detail by Pellow et al. [57] and modified by others [58]. In brief, the rats were placed at the crossing of the open and closed arms of the plus maze raised above the ground with the head facing the open arm. Testing was performed in a darkened environment. During the five-minute test, the number of entries to open and closed arms and the time spent in each arm were measured. After each rat was tested, the EPM was cleaned with a 10% ethanol solution in order to avoid interference in subsequent tests from the animal’s odors or residues. An EPM-based behavioural score was assessed as a ratio of time spent in the open arms of the maze.

### 4.6. In Vivo MRI and MRS

Imaging and spectroscopy were performed on all animals before the start of CUMS and gavage feeding, after five weeks of simultaneous stress procedure and treatment (week five), and after eight weeks of simultaneous stress procedure and treatment (week eight). Experiments were performed on a 7T MRI scanner (70/16 Pharma Scan, Bruker Biospin, GmbH, Germany) using a 72 mm transceiver RF coil (Bruker, Germany) and a 20 mm receive-only surface loop coil (Bruker, Germany). The whole scanning protocol encompassing MRI and MRS lasted approximately two hours for each animal. On imaging days, food was removed from the cage at least four hours before the examination in order to minimize the effects of food intake on neurotransmitter levels. The animals were anesthetized with an isoflurane/oxygen mixture (3.5% for induction and 1.7–2.5% for maintenance). Both respiratory rate and body temperature were continuously monitored with an MR-compatible Small Animal Monitoring System (SA Instruments, Inc., Stony Brook, NY, USA). Rats breathed freely during the MR exam and the anesthetic concentration was adjusted to maintain the respiratory rate ~50 bpm. Body temperature was maintained at 37 °C using circulating water.

The anatomical imaging was performed using three-plane T_2_-weighted rapid acquisition with a relaxation enhancement sequence (RARE, TR/TE 2500/33 ms, RARE factor 8, FOV = 30 mm × 30 mm, matrix size 256 × 256, slice thickness 1.0 mm) to serve for further positioning of R_1_ and MRS measurements.

#### 4.6.1. Longitudinal Relaxation Rate, R_1_ Measurements

In order to estimate brain water content, five single-slice inversion recovery RARE scans (TR/TE = 10 000/6 ms, TI = 30, 230, 650, 800 and 5000 ms, FOV = 30 mm × 30 mm, slice thickness = 1 mm, matrix = 64 × 64, bandwidth = 67 kHz, averages = 1, time = 2 min each) were acquired in an axial plane, covering the ventral part of the hippocampus and cerebral cortex located directly above the hippocampus.

#### 4.6.2. MRS

Magnetic field shim adjustments were performed using the built-in PARAVISION MAPSHIM routine (Bruker Biospec, Etlingen, Germany). The 2.0 × 2.0 × 5.5 mm^3^ volume of interest (VOI) was placed over the right hippocampus based on anatomical images. Proton MRS spectra were acquired over the VOI using point resolved spectroscopy (PRESS) (bandwidth 3 kHz, 4096 complex data points, TR 2.5 s, TE 16.66 ms (minimum, TE1/TE2 = 8.87/7.79 ms), 1024 averages). The echo time, TE = 16.66 ms was the minimum allowable to alleviate signal attenuation caused by J-modulation and T_2_ relaxation [59]. The water signal was suppressed using seven variable power RF pulses with optimized relaxation delays (VAPOR) [59].VAPOR inter-pulse delays and pulse amplitudes were optimized manually for each animal to achieve optimal water suppression. Non-water suppressed spectra were acquired to allow for the normalization of neurometabolites’ concentrations to the concentration of in vivo brain water.

### 4.7. MRI and MRS Data Analysis

Many studies on cerebral neurochemistry in mood disorders, both human and animals, are based on MRS [46,60,61,62,63,64,65]. The absolute quantification of metabolites levels in tissue requires appropriate calibration, and the most common practice is an internal refencing based on additional unsuppressed water MRS measurement [66]. This approach requires knowledge of the tissue water content, and together with the known molarity of pure water, the neurometabolites’ concentrations in mmol/L can be estimated. In the majority of MRS-based studies of depression it is assumed that water content level is specific for a particular tissue, e.g., in gray matter it is assumed to be 80%, in white matter it is 65%, and such values are incorporated to quantify the metabolites levels [26,67,68]. It has to be noted, however, that in long lasting depressive disorder, hippocampal atrophy is observed [24,25], in which water content decrease is also reported as measured by MRI in terms of longitudinal relaxation time T_1_ [23]. The assumption that water level is constant might lead to uncertainties when comparing metabolites between healthy and diseased cases. Complicating the issue, in longitudinal studies the water content changes due to brain maturation and aging has to be taken into account, what was shown in many MRS studies of brain development [35,36,37]. This aspect, however, is usually not taken into account in MRS studies of depressive disorders. It was shown that longitudinal relaxation rate of tissue R_1_ = 1/T_1_ is proportional to the reciprocal percentage of water content W [26]. Based on this feature, we propose to use additional measurement of R_1_ as a scaling factor for metabolites concentrations estimated from the MRS signal. Such normalization eliminates the influence of subject-dependent hydration of cerebral tissue and was used in this study, as follows.

#### 4.7.1. Water Content Change Estimation Based on R_1_ Measurements

The longitudinal relaxation rate, R_1_ (1/T_1_), maps were calculated from the RARE images as described elsewhere [69]. R_1_ values were averaged within the hippocampal area for each animal. The R_1_ is proportional to the tissue water content according to the formula [26]:

(1) $$\frac{1}{W}\sim \frac{1}{T_{1}}=R_{1},$$

where W determines the percentage water concentration in tissue. We used R_1_ as the normalization factor for further MRS data analysis to reduce the influence of between-subjects differences of hippocampal water content.

#### 4.7.2. MRS Data Analysis

MRS data was analyzed using a Java based Magnetic Resonance User Interface (jMRUI, version 6.0, MRUI Consortium, http://www.jmrui.eu, accessed on 1 February 2017) [70]. A basis set of 22 metabolites was simulated with the NMRScopeB plugin (version 2.1) [71], using the PRESS sequence parameters and magnet characteristics. The macromolecular signal, MM, was included in the basis set. The MM signal originated from our laboratory databases, and was acquired from healthy Wistar rats as described in our previous study [20]. The MRS data was then fit using this basis set. Fitting errors for each metabolite, Cramer Rao Lower Bounds (CRLB), were also evaluated using jMRUI as the standard deviation (SD) of the model from the original data and the signal-to-noise ratio (SNR) as the ratio of the maximum of the model spectrum to twice the residuals. Metabolites with CRLB > 25% were excluded from further analysis. The unsuppressed water signal was used to normalize the fitted signals of metabolites to water content of the tissue and to calculate absolute concentrations of metabolites in tissue (expressed in mM), assuming 80% water content in gray matter [27], and a water molarity of 55 mM. These data were presented as the “standard approach”. Next, the metabolites’ concentrations were corrected to individual water content by dividing the concentrations by R_1_.

### 4.8. Statistical Analyses

The statistical analyses were performed with the use of STATISTICA software (v. 13, TIBCO Software Inc., Palo Alto, CA, USA). The behavioural scores, body mass, longitudinal relaxation rates R_1_, relative water content, and absolute concentrations of metabolites were compared between groups of animals and presented as means ± standard deviation (SD), where applicable. Two-way ANOVA analysis for repeated measures was performed to assess group × time effect, followed by a post-hoc Duncan’s test for finding significant differences between groups and timepoints. When the homogeneity of variance conditions were not met (assessed by Levene test) a non-parametric one-way Friedman’s ANOVA (χ^2^ statistic) was used to assess the time effect, and one-way Kruskal-Wallis ANOVA (H statistic) with median test was applied for finding significant differences between groups at particular timepoints. When rejecting outliers, Chauvenet’s criterion was applied. *p* values lower than 0.05 were indicative of significant results [72,73].

## Figures and Tables

**Table 1:** Fitted metabolites for which Cramer Rao Lower Bounds fitting errors (CRLB,%) were lower than 25%, as estimated by jMRUI software.

METABOLITE	CRLB
Cho	2 ± 1%
Cr	3 ± 2%
GABA	5 ± 2%
Glu	2 ± 0%
Gln	3 ± 1%
GSH	5 ± 1%
MM	1 ± 0%
m-Ins	2 ± 0%
NAA	1 ± 0%
NAAG	23 ± 13%
PCr	3 ± 1%
Tau	2 ± 0%

**Table 2:** Neurometabolites’ concentrations normalized to R_1_ in JB-1 treatment and placebo groups after five and eight weeks stress protocol, with additional division of the JB-1 group according to behavioral response: “Positive response”—behavioral score > 0, “Negative response”—behavioral score = 0. Data presented as means ± SD.

Metabolites (mM*s)	After Five Weeks of Stress			After Eight Weeks of Stress		
	JB-1		Placebo (N = 9)	JB-1		Placebo (N = 9)
	Positive Response (N = 9)	Negative Response (N = 1)		Positive Response (N = 6)	Negative Response (N = 4)	
tCho	2.9 ± 0.9	2.2	2.5 ± 0.4	2.7 ± 0.3	2.2 ± 0.1 **	2.3 ± 0.3 *
tCr	15.5 ± 1.5	15.0	14.7 ± 1.5	15.3 ± 1.3	13.5 ± 1.2	14.5 ± 1.0
GABA	3.6 ± 0.7	2.6	2.9 ± 0.6	3.5 ± 0.8	2.6 ± 0.5 *	2.7 ± 0.3 *
Glu	16.2 ± 1.5	14.3	15.2 ± 1.1	16.5 ± 1.1	15.0 ± 0.9	15.1 ± 1.4
Gln	6.7 ± 0.9	7.4	6.5 ± 0.3	6.9 ± 0.7	6.0 ± 1.1	6.4 ± 0.6
GSH	2.6 ± 0.8	1.6	2.0 ± 0.3	2.2 ± 0.1	1.8 ± 0.6	1.9 ± 0.2
Gln + GSH	9.3 ± 0.9	9.0	8.4 ± 0.4 *	9.1 ± 0.9	7.8 ± 0.8 *	8.3 ± 0.6
Glx	22.9 ± 1.8	21.7	21.5 ± 1.3	23.4 ± 1.5	20.9 ± 1.9 *	21.2 ± 1.5 *
m-Ins	11.9 ± 2.2	11.7	11.0 ± 0.9	11.3 ± 0.7	10.5 ± 0.8	11.2 ± 0.8
NAA	14.4 ± 1.3	11.6	12.9 ± 1.2 *	13.9 ± 0.5	13.4 ± 0.7	13.2 ± 0.9
NAAG	0.3 ± 0.2	0.2	0.3 ± 0.1	0.3 ± 0.1	0.2 ± 0.1	0.2 ± 0.1
tNAA	14.7 ± 1.2	11.8	13.3 ± 1.2 *	14.2 ± 0.6	13.6 ± 0.8	13.2 ± 0.5 *
Tau	13.2 ± 1.7	13.3	12.1 ± 1.2	12.5 ± 1.7	11.0 ± 0.9	11.6 ± 0.7
MM	86.3 ± 12.1	124.6	86.1 ± 9.1	93.3 ± 7.1	88.2 ± 8.8	88.8 ± 10.5

means ± SD; * *p* < 0.05; ** *p* < 0.01—significant difference as compared to “Positive response” JB-1 group; After five weeks stress: t-Student test for comparison between “Positive response” JB-1 group vs. placebo group; After eight weeks stress: one-way ANOVA with post-hoc Duncan’s test for comparison between three groups: “Positive response” JB-1 vs. “Negative response” JB-1 vs. placebo.

## Data Availability

The data that support the findings of this study are available from the corresponding author upon reasonable request.

## Supplementary Materials

The following supporting information can be downloaded at: https://www.mdpi.com/article/10.3390/ijms23095173/s1.
