# Supplementary material for: Continuous Ingestion of Lacticaseibacillus rhamnosus JB-1 during Chronic Stress Ensures Neurometabolic and Behavioural Stability in Rats
Source: Int J Mol Sci. 2022 May 5;23(9):5173. doi: 10.3390/ijms23095173 (PMC9106030; doi:10.3390/ijms23095173)
Supplement: Supplementary file 1 [file ijms-23-05173-s001.zip › TABLE_S2.pdf]

Table S2. Neurometabolites' concentrations corrected to subject-dependent water content differences (normalized to R_1) with p-values in JB-1 treatment and placebo groups at baseline and after five and eight weeks stress protocol.

Metabolites (mM*s)	Baseline		After 5 weeks stress		After 8 weeks stress		p-values								
							JB-1 vs. PB			Placebo			JB-1		
	JB-1	Placebo	JB-1	Placebo	JB-1	Placebo	baseline	After 5 weeks	After 8 weeks	5 weeks vs. baseline	8 weeks vs. 5 weeks	8 weeks vs. baseline	5 weeks vs. baseline	8 weeks vs. 5 weeks	8 weeks vs. baseline
tCho	2.4 ± 0.1	2.4 ± 0.1	2.8 ± 0.3	2.5 ± 0.1	2.5 ± 0.1	2.3 ± 0.1									
tCr	14.9 ± 0.4	15.2 ± 0.3	15.4 ± 0.4	14.7 ± 0.5	14.6 ± 0.5	14.5 ± 0.3									
GABA	3.3 ± 0.2	3.4 ± 0.3	3.5 ± 0.2	2.9 ± 0.2	3.2 ± 0.2	2.7 ± 0.1		0.09	0.1			0.1			
Glu	15.9 ± 0.4	16.6 ± 0.6	16.0 ± 0.5	15.2 ± 0.3	15.9 ± 0.4	15.1 ± 0.4				0.06		**0.04**			
Gln	7.0 ± 0.1	6.5 ± 0.3	6.8 ± 0.3	6.5 ± 0.1	6.5 ± 0.3	6.4 ± 0.2									
GSH	2.1 ± 0.1	2.3 ± 0.1	2.5 ± 0.3	2.0 ± 0.1	2.1 ± 0.1	1.9 ± 0.1		0.06				**0.045**	0.07	0.06	
Gln+GSH	9.1 ± 0.1	8.8 ± 0.3	9.3 ± 0.3	8.4 ± 0.1	8.6 ± 0.3	8.4 ± 0.2		**0.01**						0.07	
Glx	22.8 ± 0.4	23.2 ± 0.8	22.8 ± 0.5	21.5 ± 0.4	22.4 ± 0.6	21.7 ± 0.6		0.09							
m-Ins	11.1 ± 0.5	10.6 ± 0.4	11.8 ± 0.6	11.0 ± 0.3	10.9 ± 0.2	11.2 ± 0.2									
NAA	14.0 ± 0.3	14.5 ± 0.5	14.1 ± 0.5	12.9 ± 0.4	13.7 ± 0.2	13.2 ± 0.3		0.07		0.06		0.1			
NAAG	0.3 ± 0.0	0.2 ± 0.1	0.3 ± 0.1	0.3 ± 0.0	0.3 ± 0.0	0.3 ± 0.0									
tNAA	14.3 ± 0.3	14.7 ± 0.5	14.4 ± 0.4	13.3 ± 0.4	14.0 ± 0.2	13.5 ± 0.3		0.07		0.1		0.06			
Tau	12.8 ± 0.3	12.9 ± 0.4	13.3 ± 0.5	12.1 ± 0.4	11.9 ± 0.5	11.6 ± 0.2		0.08				**0.02**		**0.02**	
MM	91.0 ± 3.3	101.5 ± 3.6	90.1 ± 5.0	86.1 ± 2.9	91.2 ± 2.3	88.8 ± 3.3	0.06			**0.008**		**0.02**			
