# Supplementary material for: Continuous Ingestion of Lacticaseibacillus rhamnosus JB-1 during Chronic Stress Ensures Neurometabolic and Behavioural Stability in Rats
Source: Int J Mol Sci. 2022 May 5;23(9):5173. doi: 10.3390/ijms23095173 (PMC9106030; doi:10.3390/ijms23095173)
Supplement: Supplementary file 1 [file ijms-23-05173-s001.zip › TABLE_S1.pdf]

Table S1. Neurometabolites' concentrations calculated with standard water referencing (assuming, that gray matter water content is ~80%) with p-values in JB-1 treatment and placebo groups at baseline and after five and eight weeks stress protocol.

Metabolites (mM)	Baseline		After 5 weeks stress		After 8 weeks stress		p-values								
							JB-1 vs. PB			Placebo			JB-1		
	JB-1	Placebo	JB-1	Placebo	JB-1	Placebo	baseline	After 5 weeks	After 8 weeks	5 weeks vs. baseline	8 weeks vs. 5 wks	8 weeks vs. baseline	5 weeks vs. baseline	8 weeks vs. 5 weeks	8 weeks vs. baseline
tCho	1.4 ± 0.1	1.3 ± 0.1	1.6 ± 0.2	1.5 ± 0.1	1.5 ± 0.0	1.4 ± 0.1									
tCr	8.7 ± 0.2	8.5 ± 0.1	8.8 ± 0.2	8.7 ± 0.2	8.6 ± 0.2	8.6 ± 0.2									
GABA	1.9 ± 0.1	1.9 ± 0.2	2.0 ± 0.1	1.7 ± 0.1	1.9 ± 0.1	1.6 ± 0.1						0.08			
Glu	9.2 ± 0.2	9.2 ± 0.3	9.2 ± 0.2	9.0 ± 0.2	9.4 ± 0.2	8.9 ± 0.2									
Gln	4.1 ± 0.1	3.6 ± 0.1	3.9 ± 0.2	3.7 ± 0.1	3.8 ± 0.1	3.9 ± 0.1	0.08								
GSH	1.2 ± 0.1	1.3 ± 0.1	1.4 ± 0.1	1.2 ± 0.1	1.2 ± 0.1	1.1 ± 0.0		0.09					0.09		
Gln+GSH	5.3 ± 0.1	4.9 ± 0.2	5.3 ± 0.2	5.0 ± 0.1	5.0 ± 0.1	5.0 ± 0.1		0.08							
Glx	13.3 ± 0.3	12.9 ± 0.3	13.1 ± 0.2	12.7 ± 0.2	13.2 ± 0.2	12.8 ± 0.3									
m-Ins	6.4 ± 0.2	5.9 ± 0.2	6.8 ± 0.3	6.2 ± 0.3	6.4 ± 0.1	6.7 ± 0.1						0.05			
NAA	8.1 ± 0.2	8.0 ± 0.2	8.1 ± 0.2	7.6 ± 0.2	8.1 ± 0.1	7.8 ± 0.2									
NAAG	0.2 ± 0.0	0.1 ± 0.0	0.2 ± 0.0	0.3 ± 0.1	0.2 ± 0.0	0.2 ± 0.0									
tNAA	8.3 ± 0.2	8.2 ± 0.2	8.3 ± 0.2	7.8 ± 0.2	8.2 ± 0.1	8.0 ± 0.2									
Tau	7.5 ± 0.1	7.2 ± 0.2	7.6 ± 0.3	7.1 ± 0.2	7.0 ± 0.2	6.9 ± 0.1								0.02	0.1
MM	52.6 ± 1.5	56.5 ± 1.9	51.7 ± 2.8	50.6 ± 1.3	53.7 ± 1.1	52.5 ± 1.5				0.04					
